# MiR-20a Is Upregulated in Anaplastic Thyroid Cancer and Targets *LIMK1*

**DOI:** 10.1371/journal.pone.0096103

**Published:** 2014-05-23

**Authors:** Yin Xiong, Lisa Zhang, Electron Kebebew

**Affiliations:** Endocrine Oncology Branch, Center for Cancer Research, National Cancer Institute, Bethesda, Maryland, United States of America; Sun Yat-sen University Medical School, China

## Abstract

**Background:**

There have been conflicting reports regarding the function of miR-20a in a variety of cancer types and we previously found it to be dysregulated in sporadic versus familial papillary thyroid cancer. In this study, we studied the expression of miR-20a in normal, benign and malignant thyroid samples, and its effect on thyroid cancer cells *in vitro* and *in vivo*.

**Methodology/Principal Findings:**

The expression of miR-20a in normal, benign and malignant thyroid tissue was determined by quantitative RT-PCR. Thyroid cancer cells were transfected with miR-20a and the effect on cellular proliferation, tumor spheroid formation, and invasion was evaluated. Target genes of miR-20 were determined by genome-wide mRNA expression analysis with miR-20a overexpression in thyroid cancer cells and target prediction database. Target genes were validated by quantitative PCR and immunoblotting, and luciferase assays. MiR-20a expression was significantly higher in anaplastic thyroid cancer than in differentiated thyroid cancer, and benign and normal thyroid tissues. MiR-20a significantly inhibited thyroid cancer cell proliferation *in vitro* (p<0.01) and *in vivo* (p<0.01), tumor spheroid formation (p<0.05) and invasion (p<0.05) in multiple thyroid cancer cell lines. We found that *LIMK1* was a target of miR-20a in thyroid cancer cell lines and direct knockdown of LIMK1 recapitulated the effect of miR-20a in thyroid cancer cells.

**Conclusions/Significance:**

To our knowledge, this is the first study to demonstrate that miR-20a plays a role as a tumor suppressor in thyroid cancer cells and targets *LIMK1*. Our findings suggest the upregulated expression of miR-20a in anaplastic thyroid cancer counteracts thyroid cancer progression and may have therapeutic potential.

## Introduction

Thyroid cancer is the most common endocrine cancer and one of the fastest growing cancer diagnoses in the United States [1], [2]. Thyroid cancers originate from follicular cells and parafollicular cells [3], [4]. Thyroid cancers originating from follicular cells account for over 95% of all thyroid cancer cases and are classified into four major histologic groups (papillary thyroid cancer (PTC), follicular thyroid cancer (FTC), Hürthle cell carcinoma (HCC), and anaplastic thyroid carcinoma (ATC)). MicroRNAs (miRNAs) have been shown to be dysregulated in thyroid cancers originating from follicular cells [5]–[7]. MiRNAs are small, noncoding RNAs, which are approximately 21 nucleotides long and regulate gene expression [8], [9]. Generally, miRNAs bind to the 3′-untranslated region (3′-UTR) of the target gene, leading to repressed translation or degradation of mRNA [9], [10].

MiR-20a is a member of the miR-17-92 cluster located on chromosome 13. Previous studies have shown that miR-20 may function to promote or inhibit the hallmarks of malignant cell phenotype in a cell type specific manner [11]–[13]. For example, miR-20a overexpression inhibits cellular proliferation, invasion, and tumor metastasis in breast cancer cell lines [11], [12]. On the other hand, miR-20a suppresses *E2F1* expression in human B cell line P-493-6, a transcription factor that promotes G1-S phase progression in mammalian cells [14]. This finding suggests that miR-20a function may be different depending on cell type. We previously found miR-20a to be upregulated in familial PTC as compared to sporadic cases, which are thought to be more aggressive [3], [15]. Takakura et al. [16] also found that miRNAs of the miR-17-92 cluster (miR-17-3p, -17-5p, -18a, -19a, -20a, -19b, and -92-1) were overexpressed in ATC cell lines.

In this study, we characterize the expression of miR-20a in normal, benign and malignant thyroid samples, and studied its effect on thyroid cancer cells *in vitro* and *in vivo*. We also performed analysis of miR-20a target genes using target prediction database and genome-wide expression with miR-20a overexpression, and validated the target genes with luciferase assay. Lastly, we show that LIMK1 recapitulates the effects of miR-20a in thyroid cancer cells.

## Materials and Methods

### Human thyroid tissue samples and animal experiments

Thyroid tissue samples were snap frozen at the time of thyroidectomy under a protocol approved by the Office of Human Subject Research at the National Institutes of Health Clinical Center, after written informed consent. All tissue samples underwent secondary additional histology review by an endocrine pathologist to confirm the diagnosis and identify samples with greater than 80% tumor cells. Tissue samples were classified as normal, benign (multinodular goiter, follicular adenoma, Hürthle cell adenoma), differentiated thyroid cancer [DTC] (classic PTC, follicular variant of PTC, FTC), and ATC. Normal thyroid tissue was obtained from patients undergoing thyroidectomy for benign or malignant disease from the contralateral thyroid lobe. In all, 8 ATC, 22 DTC, 24 benign, and 11 normal thyroid tissue samples were analyzed.

The National Cancer Institute Animal Care and Use Committee approved the protocols for animal care and handling in the present study. Any mouse experiencing significantly abnormal neurological signs, bleeding from any orifice, impaired mobility, rapid weight loss, debilitating diarrhea, rough hair coat, hunched posture, labored breathing, lethargy, persistent recumbence, jaundice, anemia, self-induced trauma, becomes moribund or otherwise becomes unable to obtain food or water, or with a tumor 2 cm or greater in diameter has been immediately euthanized by CO_2_ chamber.

### Cell lines and culture conditions

Human thyroid cancer cell lines XTC-1 (HCC) (kindly provided by Dr. Orlo H. Clark (San Francisco, CA)) [17], FTC-133 (FTC) (kindly provided by Dr. Peter Goretzki (Germany)), and TPC-1 (PTC) (kindly provided by Dr. Nabuo Satoh (Japan)[18]) were maintained in DMEM with 4,500 mg/L of D-glucose and L-glutamine, and 110 mg/L of sodium pyruvate, supplemented with 10% fetal bovine serum (FBS), thyroid-stimulating hormone (TSH) (10 mU/mL), penicillin (10,000 U/mL), streptomycin (10,000 U/mL), Fungizone (250 mg/mL), and insulin (10 µg/mL) in a standard humidified incubator at 37°C in a 5% CO_2_ and 95% O_2_ atmosphere. Serum-free media (DMEM-F12 media), supplemented with four hormones (insulin [10 µg/mL], somatostatin [10 ng/mL], transferrin [5 µg/mL], and hydrocortisone [0.36 ng/mL]), was used for the functional genomics studies. The human ATC cell line C643 was kindly provided by Dr. Rebecca Schweppe, with permission from Dr. N.-E. Heldin (Sweden)[18], and was maintained in RPMI (Invitrogen, Carlsbad, CA) containing 10% FBS. All of the cell lines were authenticated by short tandem repeat on October 9, 2013 and the XTC-1 cell line also was confirmed to express thyroglobulin and sodium iodine symporter as previously reported [17].

### MiRNA transfection

Mature miRNA precursor (pre-miR-20a; Applied Biosystems, Foster City, CA) was transfected into cells at a concentration of 25 nM using Lipofectamine RNAiMAX (Invitrogen, Carlsbad, CA), following the manufacturer's protocol. An oligonucleotide not representing any known miRNA (Pre-miR miRNA Precursor Molecules—Negative Control #1; Applied Biosystems, Foster City, CA) was used as a negative control.

### SiRNA transfection

LIMK1 siRNA #A (ID s8188), siRNA #B (ID s8189) and siRNA #C (ID s 8190) (Applied Biosystems, Foster City, CA) were transfected into cells at a concentration of 60 nM using Lipofectamine RNAiMAX (Invitrogen, Carlsbad, CA), following the manufacturer's protocol. Silencer Select Negative Control #1 siRNA (Applied Biosystems) was used as a negative control.

### RNA isolation and quantitative real-time RT-PCR

Total RNA was isolated from the cell lines using the TRIzol reagent (Invitrogen, Carlsbad, CA). The TaqMan MiRNA Assay (Applied Biosystems, Carlsbad, CA) was used to measure the miRNA expression level. Total RNA was reverse transcribed with a miRNA-specific primer, followed by real-time PCR with TaqMan probes. U6 was used as an endogenous control. The relative amount of mRNAs in *LIM kinase 1* (*LIMK1*) was determined using the TaqMan Assay (Applied Biosystems, Carlsbad, CA) on an ABI 7900 HT system, and human *GAPDH* was used as an endogenous control. The ΔΔ Ct method was used to calculate expression levels.

### Western blot

Whole-cell lysate was prepared with RIPA buffer (Thermo Scientific, Rockford, IL). LIMK1 protein level was determined by Western blot using a rabbit polyclonal anti-LIMK1 antibody (1∶1500 dilution; Cell Signaling Technology, Inc., Danvers, MA). GAPDH protein was detected by using a mouse monoclonal anti-GAPDH (#0411) antibody (Santa Cruz Biotechnology, Santa Cruz, CA).

### Proliferation assay

Cell proliferation was determined using the CyQUANT Cell Proliferation Assay (Invitrogen, Carlsbad, CA), according to the manufacturer's protocol. The fluorescence intensity was measured using a fluorescence microplate reader (Molecular Devices, Sunnyvale, CA), with excitation at 485 nm and emission detection at 538 nm.

### Invasion assay

Cellular invasion was measured using the BD BioCoat Matrigel Invasion Chamber (BD Biosciences, Bedford, MA), according to the manufacturer's instructions. Cell culture medium with 10% FBS was used as a chemoattractant in the lower well of the Boyden chamber. After rehydration of the basement membrane, thyroid cancer cells were seeded in the upper compartment of the chamber in serum-free medium (4×10^4^ cells per well). After incubation at 37°C in 5% CO_2_ for 22 hours, the non-invading cells were removed from the upper surface, and the cells that had invaded the membrane to the lower surface were stained with Diff-Quik Stain Set (Siemens Healthcare Diagnostics, Inc., Newark, DE). Images were taken from the membrane of each insert under a microscope (50× magnification) using a digital camera. The images were viewed on the computer screen and the cells in individual fields of each insert were manually counted. The percent of cells invading was determined by counting the number of cells invading through the Matrigel matrix and membrane relative to the number of cells migrating through the membrane of the control inserts without the Matrigel matrix. An invasion index was calculated based on the ratio of the percent of invading cells divided by the percent of invading cells of control cells.

### Spheroid culture

Two days after miRNA transfection, FTC-133 cells were trypsinized, counted, re-suspended in culture media, and plated in an Ultra Low Cluster plate (Costar, Corning, NY) at 3.5×10^4^ per well. The plates were cultured at 37°C in 5% CO_2_, and the medium was changed every 2 to 3 days. After 2 weeks of culture, cells were stained with Crystal Violet and photographed under a microscope. The total area occupied by spheroids within an image was measured by circumscribing the perimeter of each spheroid, marking the entire area, and calculating the pixel numbers using ImageJ software (Maryland, USA).

### Tumor xenograft studies

FTC-133 cells transfected with miR-20a or miR-NC were inoculated subcutaneously (10^5^ viable cells) in the left and right flanks of athymic nude mice. Tumors were measured two times a week with calipers, and volumes were calculated as length × width × height. Autopsy tumor samples were photographed to document gross morphology, and then samples were weighed.

### Migration assay

Thyroid cancer cell migration was assessed using a scratch-wound assay. 150,000 cells were transfected with miRNAs (25 nM) or siRNAs (60 nM) and were plated in six-well plates and allowed to attach and grow for 44 hours (miRNAs) or 72 hours (siRNAs). Thereafter, three vertical wounds were made with a sterile 10-µl pipette tip and a horizontal line was made across the three lines so that cells could be observed at the same point. The cells were inspected every 12 hours and measurements taken up to 24 hours.

### Genome-wide mRNA expression array

FTC-133 cells were transfected with miR-20a and miR-NC. Three days post-transfection, cells were harvested. Total RNA was extracted from cells using Trizol (Invitrogen, USA). RNA quality was ensured using the Agilent RNA 6000 Nano kit and the Bioanalyzer 2100. One-hundred fifty nanograms of total RNA was used to perform cDNA reverse transcription, synthesis, amplification, fragmentation, and terminal labeling with the GeneChip WT Sense Target Labeling and Control Reagents (Affymetrix, Santa Clara, CA). Approximately 25 ng/µL of cDNA was hybridized to the Affymetrix Human Gene 1.0 ST Array GeneChip. The arrays were washed and stained using the fluidics protocol FS450_0007 procedure on an Affymetrix Fluidics Station 450. The probe intensities were scanned with the GeneChip Scanner 3000. The raw data was normalized and analyzed using Partek Genomic Suite (Partek, Inc., St. Louis, MO). Variance analysis was used to determine the probe sets that were significantly different between the two groups. The gene list was filtered with a fold-change cutoff of 1.5, resulting in an output of significant differential expression at p≤0.05 and 1.5-fold or more differences.

### Predictions of micro-RNA targets

TargetScan 5.1 (http://targetscan.org/) was used to identify potential targets for miR-20a regulation in thyroid tissue.

### Luciferase reporter assay

The 1223 base pair 3′-UTR of human *LIMK1* was cloned into an empty luciferase reporter vector pEZX-MT01 (GeneCopoeia, Rockville, MD), generating a wild-type *LIMK1* UTR luciferase reporter construct (pEZX-LIMK1-UTR). For the dual luciferase assay, FTC-133 cells were plated in triplicate into 12-well plates and co-transfected with 0.25 µg of the reporter construct and 15 pmol of miR-20a or miR-NC by using Lipofectamine 2000 (Invitrogen). At 24 hours, the cells were lysed and assayed for both firefly and renilla luciferase using Luc-Pair miR Luciferase Assay Kit (GeneCopoeia, Rockville, MD) on a SpectraMax M5e microplate reader (Molecular Device, Sunnyvale, CA), according to the manufacturers' instructions.

### Data analysis

Data is presented as mean ± standard error of the mean. To determine statistical significance, variance analysis and t test were used, as appropriate. A p value of less than 0.05 was considered statistically significant.

## Results

### MiR-20a is overexpressed in anaplastic thyroid cancer (ATC)

**Figure 1:**
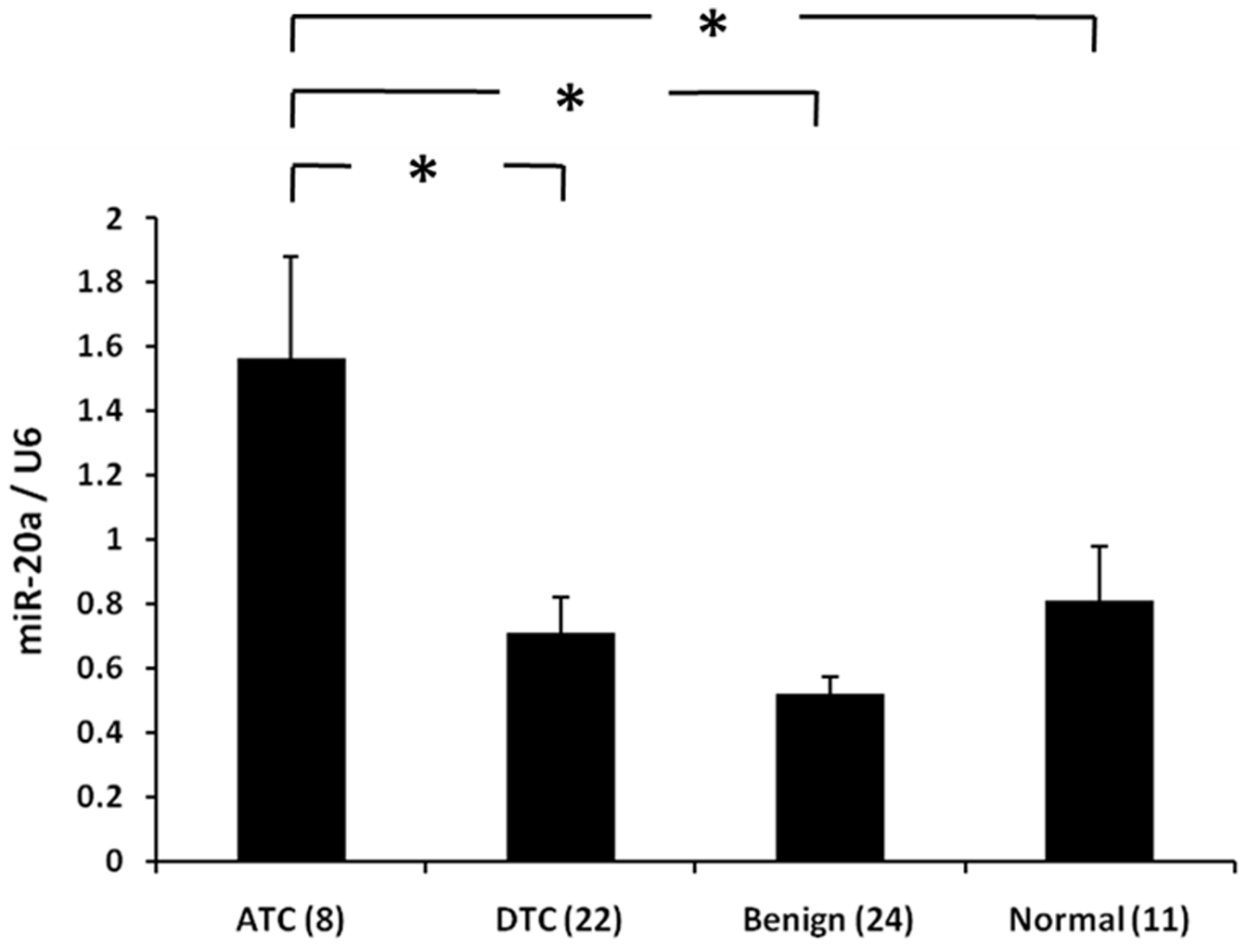
Expression level of miR-20a in human thyroid tissue samples. Y axis represents relative miR-20a expression level normalized to U6 (2^−ΔΔCt^ value). Quantitative real-time PCR was performed using total RNA from 65 human tissues (8 ATCs, 22 DTCs, 24 benign, and 11 normal). Error bars represent standard error of mean (* indicates p<0.05).

We found the expression level of miR-20a was significantly higher in ATC than in DTC, benign and normal thyroid tissues (**Fig. 1**). There was no significant difference in miR-20a expression level by *BRAF* mutation status (p = 0.62) or extent of disease (p = 0.70 for tumor size; p = 0.12 for lymph node metastasis) in DTC or PTC.

### MiR-20a regulates thyroid cancer cell proliferation, spheroid formation, and invasion

**Figure 2:**
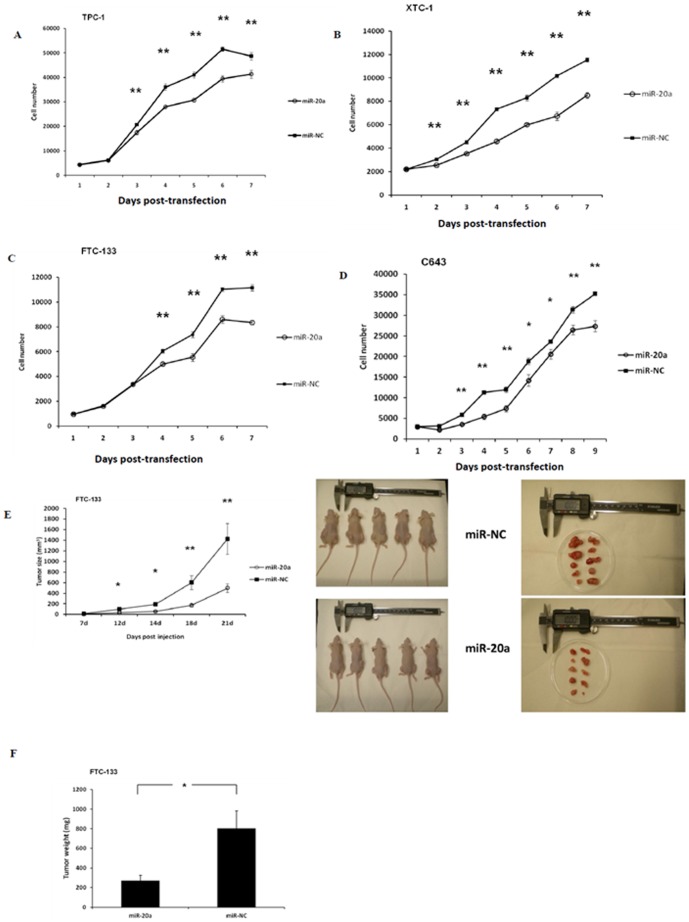
MiR-20a overexpression inhibits cellular proliferation. **(A–D)**. Thyroid cancer cell line proliferation with miR-20a overexpression. The Y axis represents the cell number. (**E–F**) Thyroid cancer *in vivo* growth, *ex vivo* tumor harvests and weight. Error bars represent standard error of mean (* indicates p<0.05; ** indicates p<0.01).

We overexpressed miR-20a in four thyroid cancer cell lines (TPC-1, XTC-1, FTC-133, and C643) using miR-NC as a negative control to determine its effect on cell proliferation. MiR-20a overexpression significantly inhibited cell proliferation by 24% in TPC-1 cells at 144 hours (p<0.001), 34% in XTC-1 cells at 144 hours (p<0.001), 22% in FTC-133 cells at 144 hours (p<0.001), and 22% in C643 cells at 216 hours (**Fig. 2A–D**). We evaluated the effect of miR-20a on tumor growth *in vivo*. We found that tumor xenografts derived from FTC-133 cells transfected with miR-20a were significantly smaller than tumor xenografts from the miR-NC group (p<0.01) (**Fig. 2E**), and the tumor weights derived from FTC-133 cells transfected with miR-20a were also significantly less than the tumor weights in the miR-NC group (p<0.05) (**Fig. 2F**).

**Figure 3:**
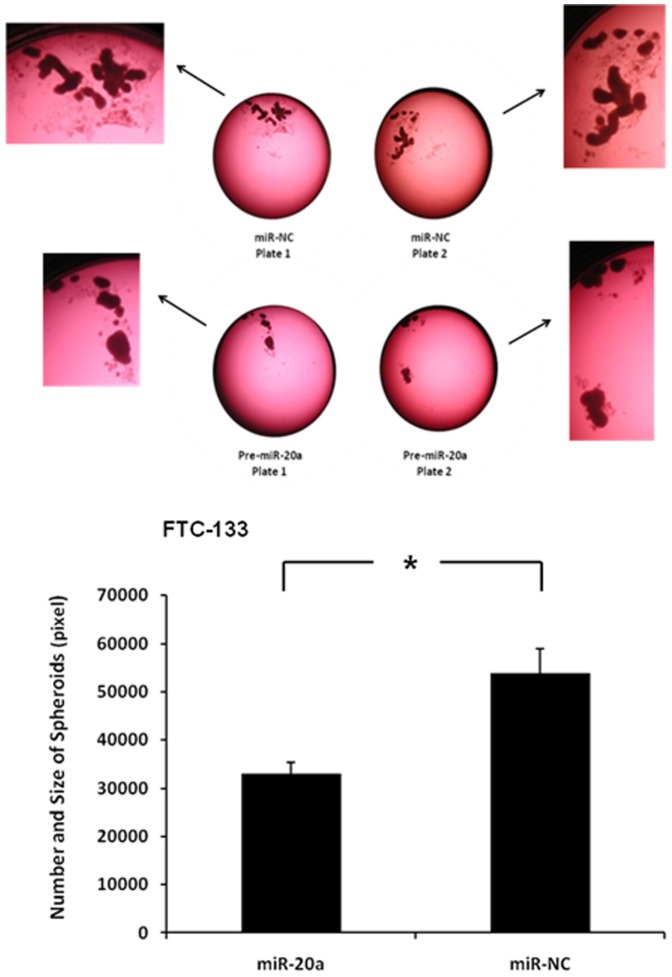
MiR-20a overexpression decreases tumor spheroids. (**A**) Representative image of spheroids in culture with miR-20a overexpression. (**B**) Quantification of spheroid difference with miR-20a overexpression. The total area occupied by the spheroids within an image was measured by circumscribing the perimeter of each spheroid, marking the entire area, and calculating the pixel numbers with ImageJ software (Maryland, USA). The Y axis represents the size and number of the spheroids. Error bars represent SEM (* indicates p<0.05).

We also studied the effect of miR-20a on thyroid cancer cell tumor spheroid formation. The FTC-133 cell line forms spheroids when cultured in ultra-low adherent culture flask and with miR-20a transfection, the number and size of spheroids were significantly decreased (**Fig. 3**).

**Figure 4:**
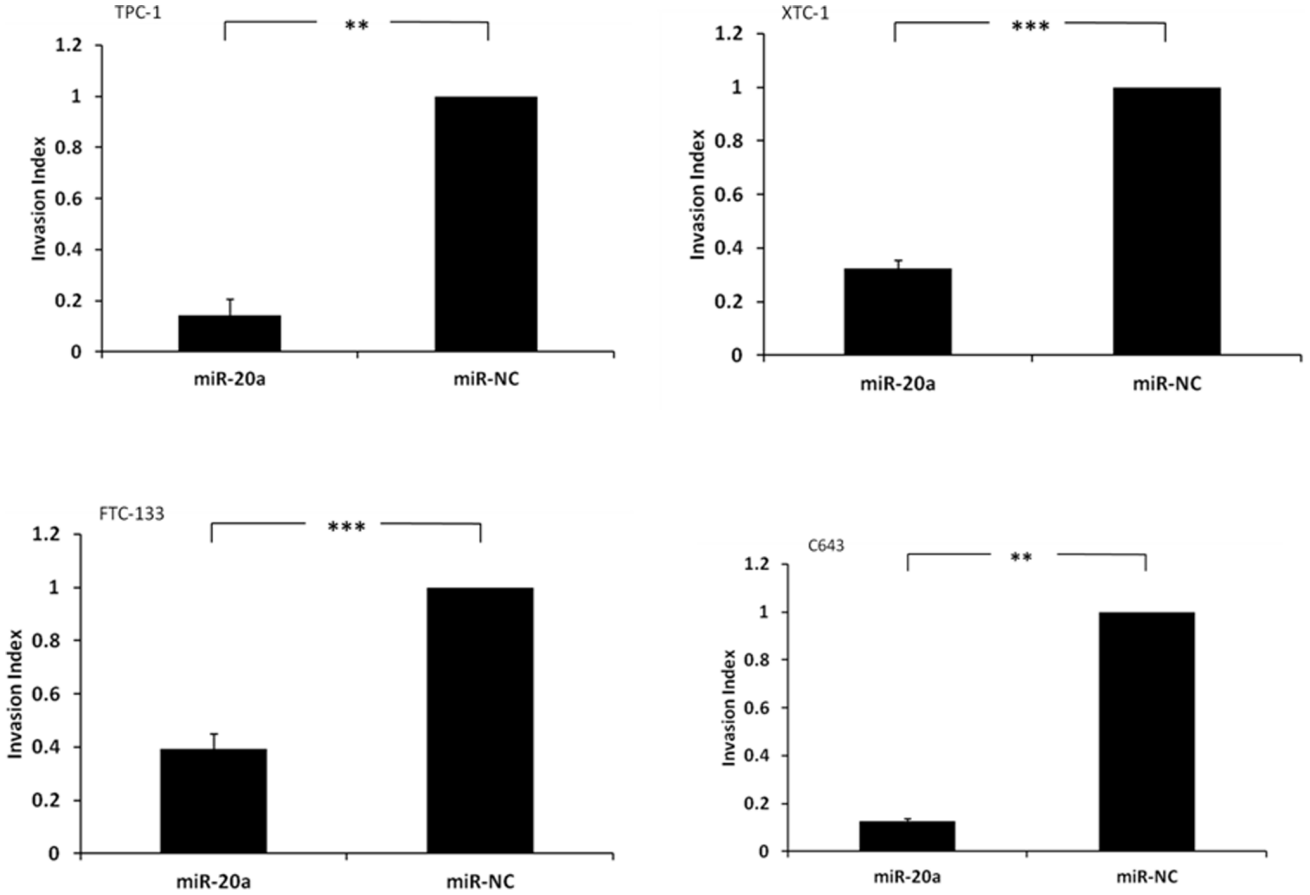
MiR-20a overexpression inhibits thyroid cancer cell invasion. (**A**) TPC-1 thyroid cancer cell line, (**B**) XTC-1 thyroid cancer cell line, (**C**) FTC-133 thyroid cancer cell line, and (**D**) C643 thyroid cancer cell line. The Y axis represents the invasion index of the thyroid cancer cells. Error bars represent standard error of mean (* indicates p<0.05; ** indicates p<0.01; *** indicates p<0.001).

MiR-20a overexpression significantly inhibited cell invasion by 85% in TPC-1 cells (p<0.01), 67% in XTC-1 cells (p<0.001), 61% in FTC-133 cells (p<0.001), and 87% in C643 cells (p<0.01) (**Fig. 4**). No significant difference was observed between cells transfected with miR-20a and miR-NC in the wound-healing assay using TPC-1 cells and FTC-133 cells.

### MiR-20a regulates LIMK1 expression in thyroid cancer cells

**Figure 5:**
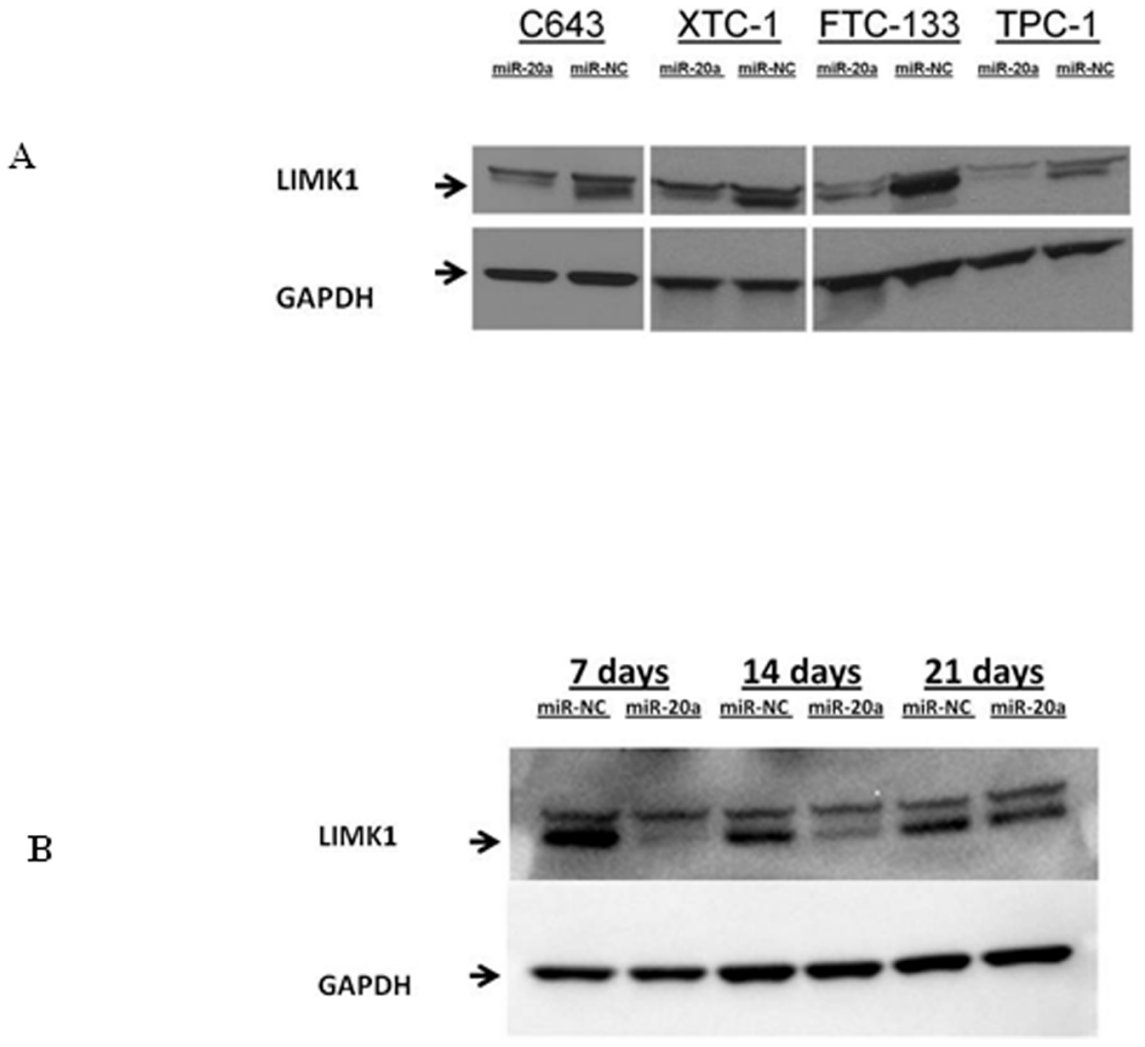
MiR-20a overexpression decreases LIMK1 protein expression in thyroid cancer cell lines. (**A**) Immunoblots for LIMK1 protein expression in C643, XTC-1, FTC-133, and TPC-1 cell lines, which were transfected with either miR-20a or miR-NC for 72 hours. (**B**) Immunoblots for endogenous LIMK1 and GAPDH in FTC-133 cells transfected with either miR-20a or miR-NC for 7 days, 14 days and 21days.

Given that miR-20a had an effect on cell proliferation and invasion *in vitro* and *in vivo*, we were interested in determining the target gene(s) of miR-20a. We used two approaches to determine miR-20a targets: (1) a target prediction database and (2) genome-wide expression analysis with miR-20a overexpression. We found 3635 predicted target genes for miR-20a using the TargetScan 5.0 software. We found 58 genes with altered expression upon miR-20a overexpression using genome-wide expression analysis. Among the 45 genes whose expression level was down-regulated, 37 genes were predicted target genes of miR-20a (**Table 1**). *LIMK1* was the most downregulated gene from this analysis and has been previously reported to have a role in tumor cell invasion and metastasis [19]–[22]. Thus, we were interested in determining whether *LIMK1* was a direct target of miR-20a. We found that LIMK1 protein expression in thyroid cancer cell lines (C643, XTC-1, FTC-133, and TPC-1) was decreased with miR-20a overexpression **(** **Fig. 5A**). The decrease in LIMK1 protein expression was observed for up to 14 days after transfection (**Fig. 5B**).

**Table 1:** Genes identified to be regulated by miR-20a using both microarray analysis and target scan analysis.*

Gene Symbol	p-value(miR-NC vs. miR-20a)	Fold-Change(miR-NC vs. miR-20a)
*LIMK1*	9.61E-07	3.78
*DAZAP2*	2.36E-06	2.78
*GNS*	2.02E-06	2.66
*MRPL24*	3.06E-06	2.57
*RNH1*	3.52E-05	2.50
*NAGK*	3.89E-05	2.29
*JAK1*	2.92E-05	2.25
*RHOC*	1.89E-08	2.12
*DPP9*	6.85E-05	2.09
*SGPL1*	5.49E-05	2.07
*CTSA*	2.18E-05	2.07
*MAP3K2*	1.29E-06	2.06
*MICA*	3.98E-06	2.04

*Genes listed were common to genome-wide gene expression analysis and target scan database, and based on change in gene expression of 2-fold or greater with adjusted p value of 0.05. In Table S1 is the entire gene list with 1.5-fold or greater change in expression, with adjusted p value of 0.05, and genes which are predicted targets by target scan analysis.

**Figure 6:**
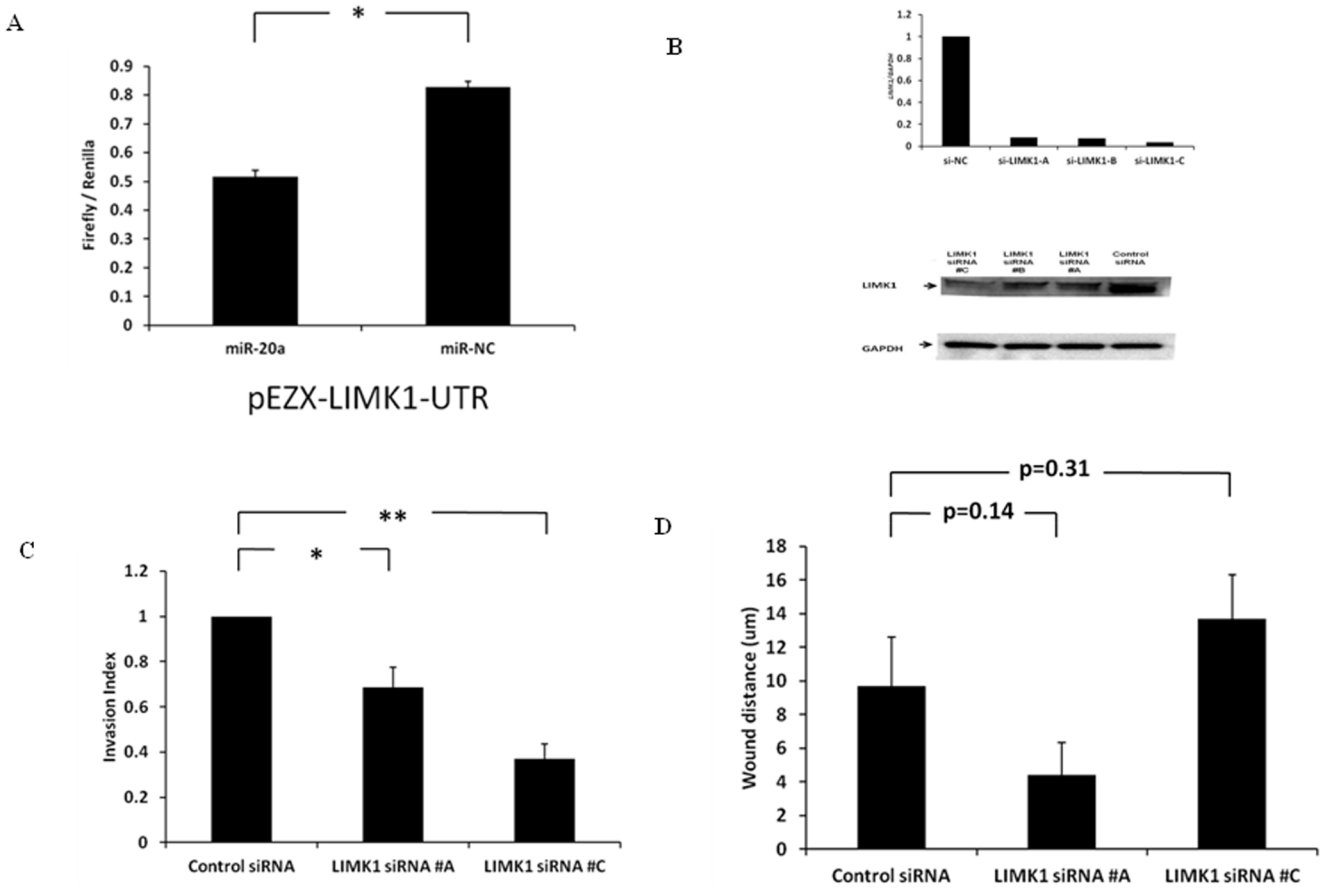
MiR-20a targets LIMK1 and LIMK1 regulates cellular invasion. (**A**) Luciferase activity of pEZX-LIMK1-UTR in FTC-133 cells when co-transfected with miR-20a or miR-NC. All luciferase measurements were made in triplicates and readings were performed at 24 hours post-transfection. Error bars represent standard error of mean (* indicates p<0.05). (**B**) *LIMK1* siRNA knockdown in FTC-133 thyroid cancer cell line. LIMK1 mRNA expression by quantitative RT-PCR (top panel). LIMK1 protein expression by Western blot (bottom panel). Data shown is for 72 hours after siRNA transfection. (**C**) Cellular invasion with LIMK1 knockdown. Transfection of *LIMK1* siRNAs inhibited FTC-133 thyroid cancer cells invasion. The Y axis represents the invasion index of the thyroid cancer cells. Data shown is for 72 hours after siRNA transfection. Error bars represent standard error of mean (* indicates p<0.05; ** indicates p<0.01). (**D**) Transfection of *LIMK1* siRNAs has no effect on the migration of FTC-133 thyroid cancer cells. The Y axis represents the wound distance. Data shown is for 72 hours after siRNA transfection. Error bars represent standard error of mean.

To determine if whether *LIMK1* was a direct target of miR-20a, we used a luciferase reporter vector pEZX-MT01 with the 3′-UTR of human *LIMK1* cloned into it, generating a *LIMK1* 3′-UTR luciferase reporter construct (pEZX-LIMK1-UTR). We performed luciferase assays with the pEZX-LIMK1-UTR (vector with 3′-UTR of LIMK1) co-transfected into the FTC-133 cell line with miR-20a or miR-NC. We found significantly decreased luciferase activity with miR-20a overexpression as compared to negative control (**Fig. 6A**), suggesting that miR-20a directly downregulates *LIMK1* expression. Given that miR-20a overexpression downregulated *LIMK1* in thyroid cancer cell lines and the most prominent effect of miR-20a on thyroid cancer cells was the inhibition of cellular invasion, we explored whether *LIMK1* has an effect on cellular invasion and migration. We found that knockdown of *LIMK1* resulted in decreased cellular invasion but not migration (**Fig. 6B and C**).

## Discussion

In this study, we found miR-20a was overexpressed in ATC as compared to DTC, benign and normal thyroid tissue. Ectopic overexpression of miR-20a significantly inhibited thyroid cancer cell proliferation *in vitro* and *in vivo*, and significantly inhibited tumor spheroid formation and invasion in multiple thyroid cancer cell lines. This suggests that miR-20a has a tumor suppressive function when it is upregulated in thyroid cancer. We also found that miR-20a regulates *LIMK1* expression, suggesting that *LIMK1* is a target gene that may mediate the suppressive effects of miR-20a on growth and invasion of thyroid cancer cells. However, one limitation of our study is the small number of ATC tumor samples analyzed but it is a rare malignancy.

MiR-20a and miR-17 (also called miR-17-5p) are located together in the miR-17-92 cluster, and they have the same seed sequence, AAAGUG. This seed sequence is shared by three other mature human miRNAs (miR-106a, -106b, and -20b), which are located in chromosome 7 and chromosome X. MiR-20a targets many genes, including *VEGFA*, *TGFBR2*, *CCND1*, *IL-8*, *MAPK14*, *PCAF*, *RUNX1*, *STAT3*, and *E2F1* [11]–[14], [23]–[26]. Many of these genes play important roles in regulating cell proliferation, cell cycle, apoptosis, and cellular migration and invasion. MiR-20a may function as either a tumor suppressor or an onco-miR, depending on the specific cell type and extracellular factors [11]–[13]. Indeed, miR-20a overexpression has been observed to inhibit cellular proliferation, invasion, and tumor metastasis in breast cancer cell lines [11], [12], suggesting a tumor suppressor role for miR-20a consistent with our data in thyroid cancer cell lines and it being upregulated in ATC (15, 16). In contrast, previous studies have shown that miR-20a may promote proliferation in human ovarian cancer cells [27], and migration and invasion in human cervical cancer cells, ovarian cancer cells, and osteosarcoma cells [27]–[29], suggesting that miR-20a functions as an onco-miR.

Takakura and associates reported that the miR-17-92 cluster (miR-17-3p, -17-5p, -18a, -19a, -20a, -19b, and -92-1) was overexpressed in ATC cell lines [16]. Using quantitative RT-PCR, they showed that miR-17-3p and miR-17-5p were overexpressed in three of six ATC tissue samples compared to normal tissue samples. They reported that transfection of inhibitors of miR-17-5p suppressed the expression level of the miR-17 family (miR-17-5p, miR-20a, and miR-106a and b) in ARO cells, resulting in cell growth reduction. However, our study of the function of miR-20a in thyroid cancer was different than the study conducted by Takakura and colleagues. First, we specifically overexpressed miR-20a to understand its effect on tumor cell biology in both undifferentiated and differentiated thyroid cancer cell lines, and we did not use inhibitors of multiple members of the miR-17-92 cluster with possible off target effects, which cannot be only selective to miR-20a. Second, we noticed that Takakura et al. used cells treated only with the transfection reagent as the negative control instead of using scrambled oligonucleotides, and we used scrambled oligonucleotides as the negative control. Additionally, the cell lines we used (C643, TPC-1, FTC-133 and XTC-1) were different than the cell lines used (ARO and FRO) by Takakura and colleagues. Lastly, the ARO cell lines used in the study by Takakura and associates may not have been authenticated thyroid cancer cell lines [18].

We found that miR-20a regulates *LIMK1* expression in thyroid cancer cell lines. *LIMK1* is regulated by the Rho signaling pathway, and it modulates actin dynamics by regulating the activity of the cofilin family proteins [30], [31]. Previous studies have shown that *LIMK1* plays a central and important role in tumor cell invasion and metastasis [19]–[22]. *LIMK1* overexpression increases the invasiveness of breast and prostate cancer cells *in vitro* and *in vivo*, and knocking down of *LIMK1* suppresses breast and prostate cancer cell invasion *in vitro* and *in vivo* [19]–[22], [32]. Based on the results from our genome-wide gene expression and target prediction analyses, we asked whether miR-20a overexpression affects *LIMK1* expression in thyroid cancer cell lines. Indeed, we found that *LIMK1* in all thyroid cancer cell lines (C643, XTC-1, FTC-133, and TPC-1) was inhibited with miR-20a overexpression, which suggests that the suppressive effect of miR-20a on cellular proliferation and invasion may be mediated by its effect on *LIMK1*. Indeed, direct knockdown of *LIMK1* had the same effects on cellular invasion and migration as observed with the overexpression of miR-20a.

Given the tumor suppressive effect of miR-20a in thyroid cancer cells we observed *in vitro* and *in vivo*, it is possible that successful delivery of miR-20a could result in tumor suppression/regression regardless of the cell type and or basal miR-20a levels [33]. The tumor suppressive effects of miR-20a could also be mediated by other genes than *LIMK1*. We validated *LIMK1* as a target because it had the lowest expression with miR-20a overexpression but as listed in **Table 1** many candidate target genes were altered with miR-20a overexpression and thus could also mediate its tumor suppressive effects.

To our knowledge, this is the first study to characterize the effect of miR-20a on thyroid cancer cell phenotypes and to show that miR-20a regulates *LIMK1* expression. Our findings suggest the upregulated expression of miR-20a in anaplastic thyroid cancer counteracts thyroid cancer progression and may have therapeutic potential [34].

## Supporting Information

Table S1 **Supplementary Table.**
(DOC)
