# Supplementary material for: MiR-20a Is Upregulated in Anaplastic Thyroid Cancer and Targets LIMK1
Source: PLoS One. 2014 May 23;9(5):e96103. doi: 10.1371/journal.pone.0096103 (PMC4032243; doi:10.1371/journal.pone.0096103)
Supplement: Table S1 — Supplementary Table. (DOC) [file pone.0096103.s001.doc]

**Table S1.** Genes identified to be regulated by miR-20a using both microarray analysis and that are predicted targets*

*Genes listed were common to genome wide gene expression analysis and target scan database, and based on change in gene expression of 1.5-fold or greater with adjusted p value of 0.05.
